# Supplementary material for: Universal control of a bosonic mode via drive-activated native cubic interactions
Source: Nat Commun. 2024 Mar 21;15:2512. doi: 10.1038/s41467-024-46507-1 (PMC10954688; doi:10.1038/s41467-024-46507-1)
Supplement: Supplementary file 1 — Supplementary Information [file 41467_2024_46507_MOESM1_ESM.pdf]

Universal control of a bosonic mode via drive-activated native cubic interactions – Supplementary Material

A. Supplementary Note 1 – Hardware design and setup

To control and measure the device, we use the microwave transceiver Presto [1]. By utilizing the second Nyquist band, Presto performs direct digital synthesis up to ~ 9 GHz with an instantaneous bandwidth of ± 500 MHz. The outputs are then filtered with external band-pass filters, which cut the unwanted signals at the other Nyquist bands. The fundamental frequency of the SNAIL-resonator was chosen such that both the $1\omega_0 = 4.1582$ and the $2\omega_0 = 8.3164$ drive pulses can be generated directly. To synthesize the $3\omega_0$ pulses, we generate one tone from Presto as a constant local oscillator at 7.9 GHz and mix it with another pulse-shaped tone in a three-port mixer ZX05-153-S+. Thereby, we have full phase control of all pulses used in the experiment. We note that the mixer is used outside of its specified IF bandwidth, which goes up to 4000 MHz, while we use an IF of around 4574.6 MHz. Investigating the resulting frequency content in a spectrum analyser showed appropriate mixing. However, the Nyqvist-band outputs from the Presto being mixed in a three-port mixer results in undesired frequency peaks, which we try to suppress with filters. More narrow band filtering could improve the $3\omega_0$ pulse generation and potentially improve the fidelity of the applied gates. The input lines are first amplified at room temperature, and then attenuated at the different stages of the cryostat, and finally filtered at the mixing chamber. The output signal is amplified by a HEMT amplifier at 4 K, and two low-noise amplifiers at room temperature before being digitized by Presto. The full wiring diagram is illustrated in Supplementary Figure. 1.

The system parameters are displayed in Supplementary Table I. The thermal population gets considerably enhanced by noise from the flux line amplifiers. To mitigate this, we use a microwave switch ADRF5024 which cuts the line when flux pulsed are not sent. With the switch, we are able to suppress the SNAIL-resonator thermal population to 2.4%. The population is characterized by the ratio between a SNAIL-resonator Fock level selective pulse on Fock 1 compared to Fock 0. The thermal population could potentially be decreased further by optimizing the duration during which the switch is closed.

The dispersive shift between the SNAIL-resonator and the qubit is 2.34 MHz which allows for SNAIL-resonator Fock-level selective pulses on the qubit as well as Wigner tomography, which utilizes Fock level inselective pulses.

Supplementary Table I. System parameters

Parameter	Symbol	Value
SNAIL-resonator frequency	$\omega_r/2\pi$	4.1582 GHz
SNAIL-resonator relaxation time	T_{1c}	28(3) µs
SNAIL-resonator coherence time	T_{2c}^*	2.80(8) µs
SNAIL-resonator broad-band phase noise	S_{BB}	3.6×10^{-5} ns
SNAIL-resonator $1/f$ phase noise	$\sqrt{A_{1/f}}$	4.25×10^{-5}
SNAIL-resonator thermal pop.	P_c	2.4%
SNAIL-resonator impedance	Z	57.94 Ω
SNAIL-resonator-qubit disp. shift	$\chi_{cq}/2\pi$	2.34 MHz
SNAIL-asymmetry	β	0.097
SNAIL large junction Josephson energy	E_{J}	245 GHz
Free resonator frequency	$\omega_\infty/2\pi$	8.99 GHz
Resonator 3rd order non-linear term at Kerr-free point	g_3^{dc}	−15.0 MHz
Resonator 4th order non-linear term at Kerr-free point	g_4^{dc}	−2.48 MHz
Resonator 5th order non-linear term at Kerr-free point	g_5^{dc}	14.4 kHz
Resonator 6th order non-linear term at Kerr-free point	g_6^{dc}	1.06 kHz
Resonator 2nd order AC non-linear term at Kerr-free point	g_2^{ac}	−167 MHz
Resonator 3rd order AC non-linear term at Kerr-free point	g_3^{ac}	38.2 MHz
Qubit frequency	$\omega_q/2\pi$	5.2891 GHz
Qubit relaxation time	T_{1q}	14(5) µs
Qubit decoherence time	T_{2q}^*	9.1(3) µs
Qubit anharmonicity	$\alpha_q/2\pi$	236 MHz
Qubit thermal pop.	P_q	1.3%
Readout frequency	$\omega_{ro}/2\pi$	6.8087 GHz

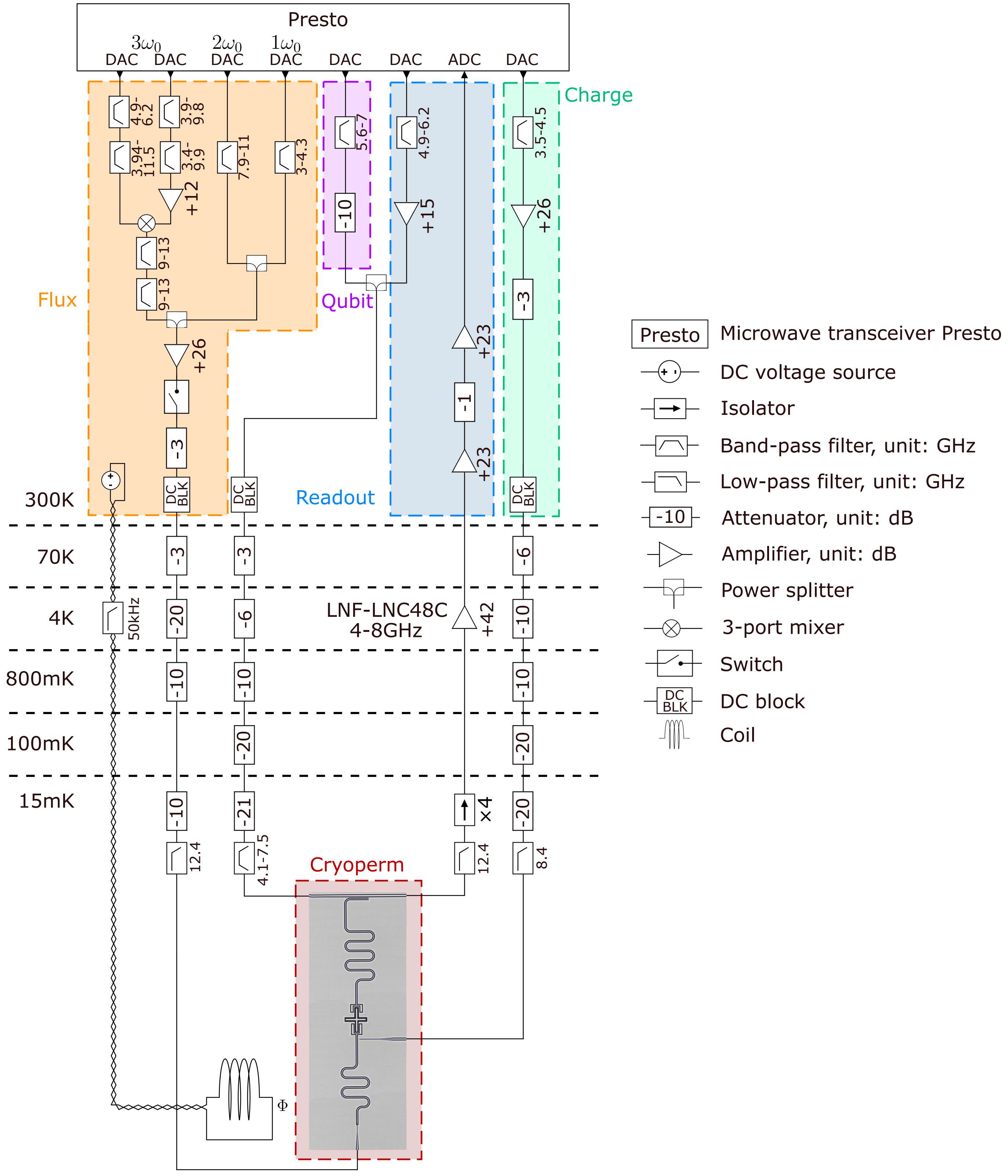

Supplementary Figure 1. Hardware setup of the experiment.

B. Supplementary Note 2 – Harmonic analysis

In this section, we explicitly derive the ω_0 and Φ parameters, respectively the SNAIL-resonator bare frequency and the participation of the SNAIL flux to the resonance respectively. They are obtained by harmonic analysis of the SNAIL-terminated resonator system. To do so, we first neglect all $n > 2$ non-linear terms in the SNAIL potential Taylor series, which amounts to replacing the SNAIL by a linear inductor of inductance

$$1/L_\mathrm{J} = E_\mathrm{J}\left(\beta\cos\varphi_\mathrm{m} + \frac{1}{n}\cos\left(\frac{\phi_\mathrm{e}-\varphi_\mathrm{m}}{n}\right)\right) \tag{1}$$

at the current anti-node of the $\lambda/4$ resonator. To treat the boundary condition, one can discretize the resonator as a series of elementary LC-oscillators, with phases labeled from φ_0 at the SNAIL boundary to φ_N at the open edge, as shown on Supplementary Figure 2. the corresponding Lagrangian is [2, 3]

$$\mathcal{L} = \frac{1}{2}\sum_{i,j=0}^{N} C\dot{\varphi}_i^2 - \frac{1}{L}\varphi_i M_{ij}\varphi_j, \qquad M = \begin{bmatrix} 1+\frac{L}{L_\mathrm{J}} & -1 & & \\ -1 & 2 & -1 & \\ & & \ddots & \\ & & & 1 \end{bmatrix}. \tag{2}$$

We diagonalize the model by $M_{ij} = \sum_k P_{ik}\lambda_k^2 P_{kj}$, and change the variables as $\sum_j P_{kj}\varphi_j = \phi_k$, to reach a Lagrangian of non-interacting modes. It is Legendre transformed to reach the Hamiltonian

$$\mathcal{H} = \sum_{k=0}^{N}\frac{1}{2C}n_k^2 + \frac{\lambda_k^2}{L}\phi_k^2. \qquad (n_k = C\dot{\phi}_k) \tag{3}$$

We perform canonical quantization and introduce creation/annihilation operators as $\hat{a}_k = (\hat{\phi}_k + iZ_k\hat{n}_k)/\sqrt{2Z_k}$, where $Z_k = \sqrt{L/C}/\lambda_k$ and $\omega_k = \lambda_k/\sqrt{LC}$. The resulting Hamiltonian is simply

$$\hat{H} = \sum_{k=0}^{N}\omega_k\hat{a}_k^\dagger\hat{a}_k + U_\mathrm{NL}(\hat{\varphi}_0), \qquad \hat{\varphi}_0 = \sum_{k=0}^{N} P_{k0}\sqrt{\frac{Z_k}{2}}(\hat{a}_k^\dagger + \hat{a}_k), \tag{4}$$

where we reintroduced the non-linear part of the SNAIL potential $U_\mathrm{NL}(\hat{\varphi}_0)$. We now focus on the fundamental mode by truncating all sums to $k = 0$. Our goal is to express λ_0 and P_{00} in terms of ϕ_e and other circuit parameters. This is obtained by explicit diagonalization of M (*cf.* Eq. (2)) in the continuum limit, which consists of $N \to \infty$ and $L, C \to 0$, while $v = d/(N\sqrt{LC})$ the wave velocity, and $Z = \sqrt{L/C}$ the resonator impedance, are kept constant.

The diagonalization is helped by introducing a plane wave ansatz, $P_{lj} = N_k\cos(jk_l + \delta_l)$. Injecting it in the eigenvalue equation, we obtain three conditions,

$$\lambda_l \simeq k_l, \quad \tan\delta_l = \frac{L}{L_\mathrm{J}}\frac{1}{k_l}, \quad \tan(Nk_l + \delta_l) = 0. \tag{5}$$

They correspond respectively to the dispersion relation and left and right boundary conditions. We deduce the implicit equation for the fundamental frequency ω_0,

$$\frac{\omega_0}{\omega_\infty} = \frac{2}{\pi}\arctan\left(\frac{Z}{L_\mathrm{J}\omega_0}\right), \quad \omega_\infty = \frac{\pi}{2}\frac{v}{d}, \tag{6}$$

where ω_∞ is the mode frequency in the absence of the SNAIL, $1/L_\mathrm{J} \to \infty$. This equation is solved numerically to obtain ω_0. Note that Z and E_J do not appear independently in this equation. They rather form a single free parameter Z/E_J.

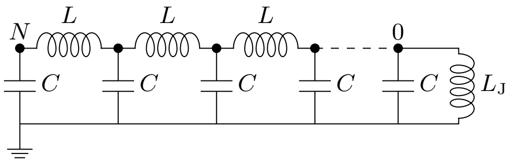

Supplementary Figure 2. Discretized, linearized model of the SNAIL-resonator.

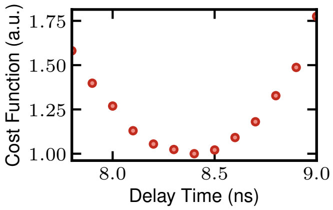

Supplementary Figure 3. Time-delay calibration of the 1ω flux and charge drives.

The last unknown is the normalisation N_0. It is obtained from the orthogonality condition $\sum_i P_{ki}P_{ki} = 1$, which provides in the continuum limit

$$N_0^{-2} = N \int_0^1 \mathrm{d}x \cos^2\left(x \frac{\pi}{2}\frac{\omega_0}{\omega_\infty} + \delta_0\right), \tag{7}$$

which is integrated into

$$\hat{\varphi}_0 = \sqrt{\frac{Z}{\pi\omega_0/\omega_\infty}\frac{1+\cos(\pi\omega_0/\omega_\infty)}{1+\mathrm{sinc}(\pi\omega_0/\omega_\infty)}}(\hat{a}_0^\dagger + \hat{a}_0). \tag{8}$$

We have reached Φ defined in Methods section A. Note that in the main text, the mode index is dropped.

C. Supplementary Note 3 – Pulse calibration

The $1\omega_r$ flux and charge pulses need to be carefully calibrated to capture the third order cross terms while avoiding the linear displacement. To calibrate the pulses, we apply them simultaneously in (close to) opposite directions with (close to) equal strengths. If the pulses are ill timed, the vacuum will displace (by the pulse arriving first) to large amplitudes where it gets distorted by higher order Kerr corrections before the other pulse displaces the state back to the origin. The cost function is the normalized mean squared distance of the measured Wigner pixels with respect to the vacuum state, which is optimal when the two pulses are timed at 8.4ns (Supplementary Figure 3).

---

[1] M. O. Tholén, R. Borgani, G. R. Di Carlo, A. Bengtsson, C. Križan, M. Kudra, G. Tancredi, J. Bylander, P. Delsing, S. Gasparinetti, and D. B. Haviland, Measurement and control of a superconducting quantum processor with a fully integrated radio-frequency system on a chip, Review of Scientific Instruments **93**, 104711 (2022).
[2] U. Vool and M. Devoret, Introduction to quantum electromagnetic circuits, Int. J. Circuit Theory Applied **45**, 897 (2017).
[3] S. Rasmussen, K. Christensen, S. Pedersen, L. Kristensen, T. Bækkegaard, N. Loft, and N. Zinner, Superconducting Circuit Companion—an Introduction with Worked Examples, PRX Quantum **2**, 040204 (2021).
